# Supplementary figures and images for: The landscape of alternative splicing in granulosa cells and a potential novel role of YAP1 in PCOS
Source: PLoS One. 2024 Dec 13;19(12):e0315750. doi: 10.1371/journal.pone.0315750 (PMC11642958; doi:10.1371/journal.pone.0315750)

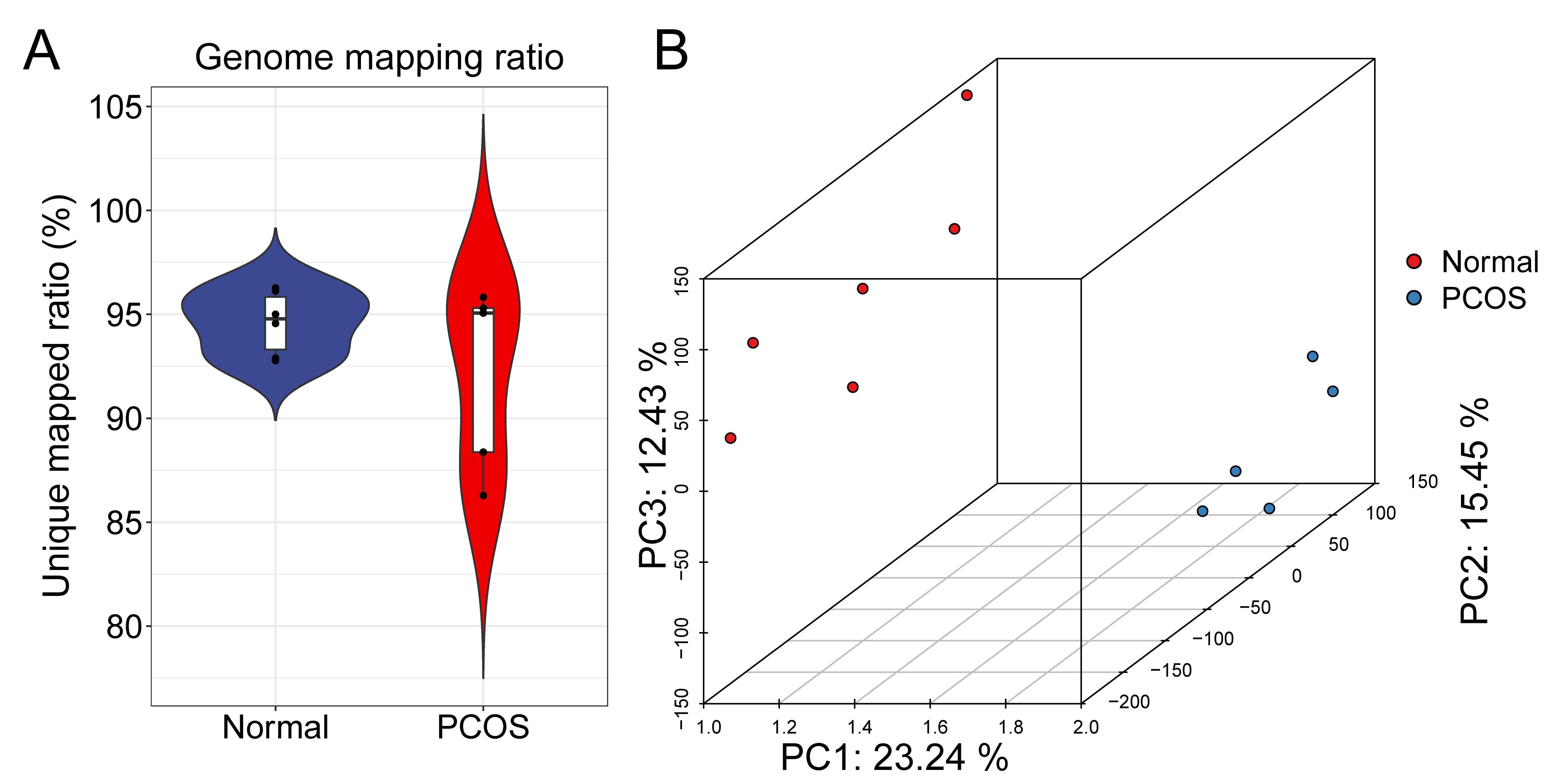

Supplement: S1 Fig — (A) Unique mapped ratio of samples between Normal and PCOS. (B) Principal component analysis of samples between Normal and PCOS. (TIF) [file pone.0315750.s001.tif]

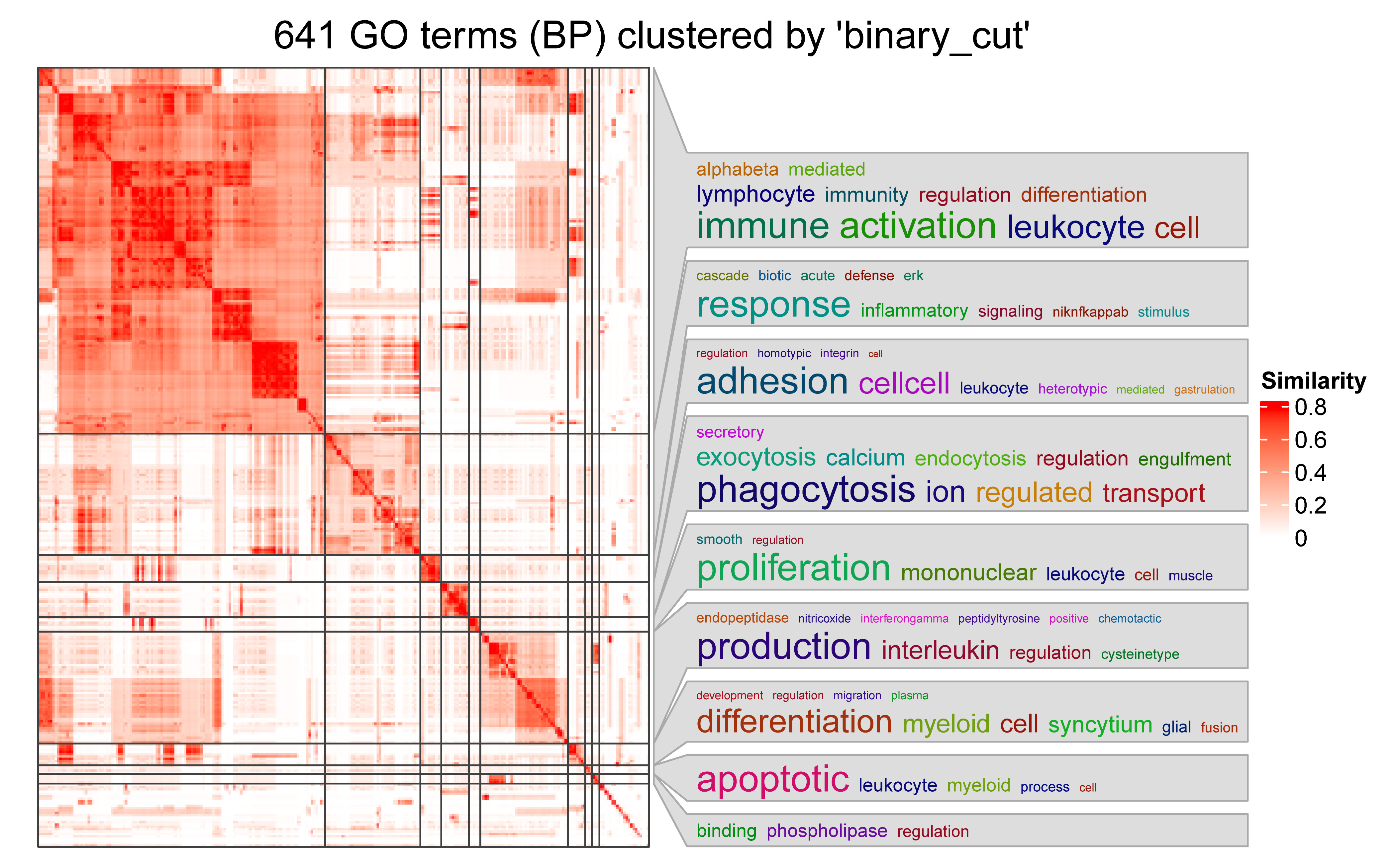

Supplement: S2 Fig — The cluster of BP terms is enriched based on differentially expressed genes. (TIF) [file pone.0315750.s002.tif]
